# Identifying Suitable *Listeria innocua* Strains as Surrogates for *Listeria monocytogenes* for Horticultural Products

**DOI:** 10.3389/fmicb.2019.02281

**Published:** 2019-10-09

**Authors:** Vathsala Mohan, Reginald Wibisono, Lana de Hoop, Graeme Summers, Graham C. Fletcher

**Affiliations:** Food Safety and Preservation Team, The New Zealand Institute for Plant & Food Research Limited, Auckland, New Zealand

**Keywords:** *Listeria* (*L.*) *monocytogenes*, *Listeria innocua*, sanitizer, UV-C, heat

## Abstract

A laboratory-based study testing 9 *Listeria innocua* strains independently and a cocktail of 11 *Listeria monocytogenes* strains was carried out. The aim was to identify suitable *L. innocua* strain(s) to model *L. monocytogenes* in inactivation experiments. Three separate inactivation procedures and a hurdle combination of the three were employed: thermal inactivation (55°C), UV-C irradiation (245 nm), and chemical sanitizer (Tsunami^TM^ 100, a mixture of acetic acid, peroxyacetic acid, and hydrogen peroxide). The responses were strain dependent in the case of *L. innocua* with different strains responding differently to different regimes and *L. innocua* isolates generally responded differently to the *L. monocytogenes* cocktail. In the thermal inactivation treatment, inactivation of all strains including the *L. monocytogenes* cocktail plateaued after 120 min. In the case of chemical sanitizer, inactivation could be achieved at concentrations of 10 and 20 ppm with inactivation increasing with contact time up to 8 min, beyond which there was no significant benefit. All *L. innocua* strains except PFR16D08 were more sensitive than the *L. monocytogenes* cocktail to the hurdle treatment. PFR16D08 almost matched the resistance of the *L. monocytogenes* cocktail but was much more resistant to the individual treatments. A cocktail of two *L. innocua* strains (PFR 05A07 and PFR 05A10) had the closest responses to the hurdle treatment to those of the *L. monocytogenes* cocktail and is therefore recommended for hurdle experiments.

## Introduction

*Listeria monocytogenes* is a Gram-positive facultative anaerobe that is found in a range of natural environments (including soil, water, and vegetation), in food-processing environments and in ready-to-eat (RTE) food products. Ingestion of *L. monocytogenes* can cause serious illness in pregnant women, neonates, and elderly and immune-compromised individuals (Food and Drug Administartion [FDA], 2003). Its ability to grow at a broad range of temperatures from 4 to 45°C (Ramaswamy et al., 2007), in salt at concentrations up to 10%, and a pH range from 4.1 to 9.6 makes *L. monocytogenes* a very significant and robust foodborne pathogen (Buchanan et al., 2017).

Control measures including physical and chemical treatments have greatly reduced the prevalence of *L. monocytogenes* in a variety of food products and in food-processing environments (Cruz and Fletcher, 2012; Stollewerk et al., 2016). However, the human disease incidence rate has not decreased over the past decades (Lebreton et al., 2016; Buchanan et al., 2017). The incidence of human listeriosis cases caused by *L. monocytogenes* averages at around 1 per 200,000 people in New Zealand, with an estimated 84.9% of cases being food related (MPI, 2013). Although regulatory controls and industry actions have been in place for many years in the United States, listeriosis outbreaks from dairy products showed no decrease in frequency (Cartwright et al., 2013), and outbreaks from fresh horticultural products have been a concern in the past decade (Ponniah et al., 2010; Cartwright et al., 2013; Chen et al., 2016; Wadamori et al., 2017). Food safety criteria for *Listeria monocytogenes* in RTE foods was implemented in 2006 in Europe; however, an increasing trend was noticed in human invasive listeriosis over the period 2009–2013 in the European Union and European Economic Area (EU/EEA). The notification rates increased rapidly with age over 65 years and predominantly in males and there were 12 outbreaks in 2013 (1,615, 1,663, 1,515, 1,644, 1,763 clinically confirmed cases recorded in 2009, 2010, 2011, 2012, and 2013, respectively) (EFSA Panel on Biological Hazards [BIOHAZ], Ricci et al., 2018). Fresh produce is often eaten raw so the high temperatures that are used to eliminate pathogens from other food products cannot be used, meaning that other control strategies must be found.

Because of the pathogenicity and the environmentally persistent nature of *L. monocytogenes*, it is challenging to safely conduct large-scale experiments using *L. monocytogenes* in research pilot plants or commercial settings. A surrogate bacterium is often sought that has similar genotypic as well as phenotypic characteristics as using surrogates gives a safety margin to protect researchers by preventing exposure to pathogens (Fairchild and Foegeding, 1993; Murphy et al., 2001, 2003). *L. monocytogenes* and *L. innocua* are genetically similar and until 1981 the two were not recognized as separate species (Seeliger, 1981). Since then, comparative genomic studies have differentiated hundreds of strain-specific genes for these two bacterial species (Glaser et al., 2001).

*Listeria innocua* is a non-pathogenic *Listeria* spp. found in similar environments to *L. monocytogenes*. The main phenotypic characteristic that distinguishes it from *L. monocytogenes* is that it is not hemolytic (Bille et al., 1992; Murray et al., 1999; Allerberger, 2003). Enhanced hemolytic activity testing, also known as the Christie, Atkins, Munch-Petersen (CAMP) test has been employed regularly for differentiating *L. innocua* from *L. monocytogenes* (McKellar, 1994; Capita et al., 2001; Gasanov et al., 2005). However, some strains of *L. monocytogenes* have also been shown to be non-hemolytic (Holt et al., 1994). Apart from some studies on heat inactivation in milk and meat (O’Bryan et al., 2006; Friedly et al., 2008), there have been no studies on selecting suitable strains of *L. innocua* as surrogate organisms to investigate non-thermal inactivation procedures. Although strains of *L. innocua* have been used as surrogates (Sommers et al., 2009), a knowledge gap is that, to our knowledge, no study has yet assessed whether *L. monocytogenes* and *L. innocua* behave similarly under non-thermal inactivation. Regarding previous thermal inactivation procedures, a study in hamburger patties identified *L. innocua* strains M1 and SLCC5640 to be good thermal processing surrogate models for *L. monocytogenes*. Furthermore, in that study, *L. innocua* M1 was identified as the preferred surrogate as it is more thermo-tolerant than *L. monocytogenes* and can provide a margin of safety in the evaluation for the effectiveness of heat treatments for *L. monocytogenes* (Friedly et al., 2008). Thermo-tolerance studies are typically conducted at temperatures of 60°C or higher (e.g., Friedly et al., 2008) but such temperatures cannot be applied to fresh produce. Temperatures of 55°C or lower can be applied for short periods to fruit surfaces without heating or damaging the flesh of the fruit and, as contamination by *Listeria* typically only occurs on the surface, such thermal treatments have potential as an inactivation process. However, a study of treatments for fresh-cut lettuce found that mild heat treatment (50°C) could actually increase the growth of *L. monocytogenes* during storage (Li et al., 2002). Other non-thermal inactivation procedures include application of ultraviolet (UV) radiation. UV irradiation as a means of disinfection of food products is considered to be cheap and clean, that is it leaves no chemical residues and microbes. It can be used in combination with other disinfection processes to ensure the safety of products (Chang et al., 1985; Gurzadyan et al., 1995; Montgomery and Banerjee, 2015). UV radiation is electromagnetic radiation in the wavelength range of 100–400 nm, shorter than that of visible light (400–700 nm), while longer than x-rays (<100 nm) (Dai et al., 2012). UV-C irradiation ranges between 200 and 280 nm (Vázquez and Hanslmeier, 2006). It damages bacterial and viral genetic material (Chang et al., 1985).

Considering that the main aim of this study was to select suitable *L. innocua* surrogate(s), thermal, non-thermal, and a combination of treatments (hurdle) technology were employed in the selection of surrogates. Although various strains of *L. innocua* have been used as surrogates in experimental treatments for inactivation of *L. monocytogenes*, few have been validated against *L. monocytogenes* (Friedly et al., 2008). There are none that perfectly match *L. monocytogenes* and none that have been tested against the range of treatment regimes, that the horticultural industries are interested in. The suitability of a potential surrogate varies depending on the *L. innocua* strain, food matrices, and parameters used for testing (O’Bryan et al., 2006). Here, this study reports a laboratory-based study conducted on different *L. innocua* and *L. monocytogenes* strains isolated in New Zealand from different sources, in order to select suitable *L. innocua* candidates to be used as surrogates in horticultural produce using thermal, non-thermal (UV and sanitizer) treatments, and their combinations (hurdle technology).

## Materials and Methods

### Bacterial Cultures

**FIGURE 1:**
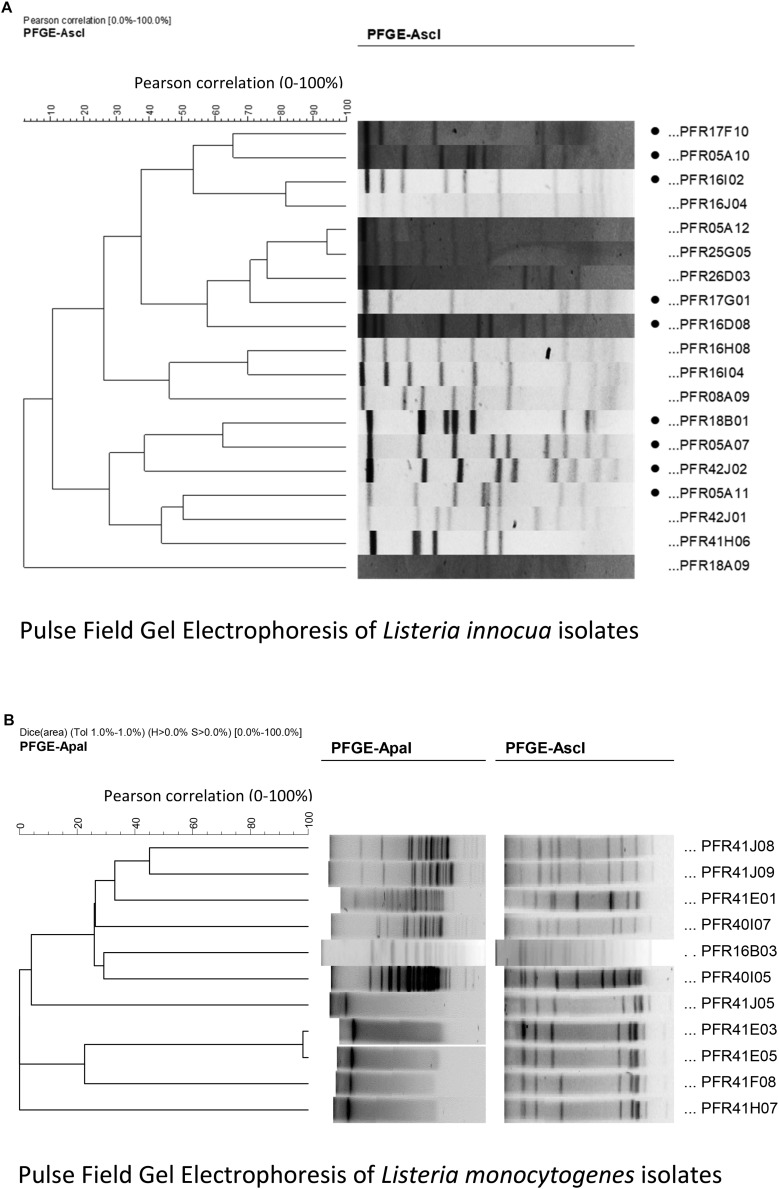
Cluster analysis of Pulse Field Gel Electrophoresis patterns performed in InfoQuest^TM^ FP software: **(A)** using *Asc*I for non-pathogenic *Listeria innocua* strains. The selected isolates of *L. innocua* are indicated with black dots in front their names; **(B)** using *Apa*I and *Asc*I restriction enzymes for *L. monocytogenes* strains used in the *LM* cocktail.

Based on their pulse field gel electrophoresis (PFGE) pulsotypes, genetically diverse *Listeria* strains were selected from The New Zealand Institute of Plant & Food Research Ltd. (PFR) Culture Collection. Pure bacterial cultures were revived from −80°C in tryptic soy broth plus 0.6% yeast extract (TSBYE, Bacto^TM^, BD, Sparks, MD, United States). Cultures were plated onto TSAYE agar (TSBYE plus 1.5% agar, Bacto^TM^, BD, Sparks, MD, United States) and incubated for 48 h at 37°C. From these plates, 3 mm diameter colonies were selected [measured using a digital vernier calliper (Model 071701, ROK International Industry, China)] and inoculated into TSBYE. Cultures were incubated for 48 h at 37°C allowing the cultures to reach the stationary phase. *L. innocua* strains from diverse New Zealand sources (*n* = 19) were typed using *Asc*I. The restriction patterns were analyzed using the InfoQuest^TM^ FP molecular analytical software tool (Bio-Rad, United States) and the similarity indices were used to build the dendrogram (Figure 1A) to analyze the genetic similarity and/or dissimilarity. Good resolution of the *L. innocua* isolates was found with just one enzyme. To conduct the lab-based pilot study, nine genetically diverse (genetic dissimilarity of at least 35% on the dendrogram) *L. innocua* strains (PFR 05A07, PFR 05A10, PFR 05A11, PFR 16D08, PFR 16I02, PFR 17F10, PFR 17G01, PFR 18B01, and PFR 42J02) were selected. *L. monocytogenes* strains from New Zealand horticultural sources (*n* = 10) were characterized by PFGE, using two rare-cutting restriction enzymes, *Asc*I and *Apa*I, and analyzed using the InfoQuest software. Ten *L. monocytogenes* strains (Figure 1B) were selected based on their genetic diversity and on the different New Zealand horticultural sources that they were isolated from (PFR 41E01, PFR 41E03, PFR 41E05, PFR 41F08, PFR 41H07, PFR 40I05, PFR 40I07, PFR 41J05, PFR 41J08, and PFR 41J09). The *L. innocua* strains were individually compared to a cocktail of the 10 strains of *L. monocytogenes* plus a well-characterized clinical ATCC reference *L. monocytogenes* strain (Scott A, PFR 16B03) (*LM* cocktail). The *LM* cocktail was prepared by mixing 1 mL of stationary phase culture from each of the *L. monocytogenes* strains. Counts of the cultures after the 48-h incubation period averaged 6.5 log_10_ MPN/mL and the OD of the *LM* cocktail was between 0.5 and 0.6 measured on PD-303 spectrophotometer (Apel, Co. Ltd., Japan). Each experiment assessed the effect of the inactivation treatments against each of the 10 *L. innocua* strains and the *LM* cocktail against their respective untreated control bacterial cultures.

### *Listeria* Quantification

All bacterial counts were enumerated using the Most probable number (MPN) technique for *Listeria* according to the method of the Food and Drug Administration (FDA) Bacterial Analytical Manual (Blodgett, 2010; Hitchins et al., 2011). Cultures were serially diluted in triplicate in 96-well plates up to 10^–15^. These plates were incubated for 48 h at 30°C after which aliquots of 2 μl were plated onto ChromAgar (750006, Paris) *Listeria.* Plates with typical growth characteristics (green colonies for *L. innocua* and green with a halo for the *LM* cocktail) were recorded for enumeration using the FDA MPN method spreadsheet (Blodgett, 2010).

### UV Treatment

The UV-C treatment was conducted in the biosafety level II cabinet equipped with two lamps, a Philips UV lamp (TUV 30 watt, G30 T8, Philips, Netherlands) and a Sankyo Denki UV germicidal lamp (G15T8, Sankyo Denki, Japan) installed side by side in the cabinet. UV-C irradiance measurements (μW/cm^2^) were carried out using a UM-10 Konica Minolta UV radiometer. To obtain different doses of UV-C light, exposure plates of cultures were placed at different distances (55, 45, 34, and 26 cm) from the UV-C lamp for 20 min. To calculate the UV-C dose, the radiometer was left in the cabinet at the respective distances and UV-C irradiance (μW/cm^2^) was recorded at 15 s intervals. UV-C doses after 20 min exposure at the respective distances from the lamp were 600, 672, 1009, and 1346 mJ/cm^2^. For one of the hurdle treatments described below, cultures were placed 45 cm from the lamp for just 10 min which gave a dose of 328 mJ/cm^2^.

Ten microliters of 48-h *L. innocua* cultures or the *LM* cocktail were dispensed into individual wells in 12-well plates (Corning-Costar, CLS3512, supplied by Sigma–Aldrich). The plates were exposed to UV-C light for 20 min. Immediately after UV-C treatment, 490 μl of Buffered *Listeria* Enrichment Broth (BLEB, Acumedia, United States) was added into each well, and thoroughly mixed by repeated pipette aspiration. These suspensions were transferred into a sterile 96-well plate (Greiner Bio-One, GmbH, Frickenhausen, Germany) and survivors quantified using the MPN method.

### Sanitizer Treatment

Tsunami^TM^ 100 (Ecolab Inc., MN, United States) was diluted to working concentrations of 10, 20, 40, and 80 ppm, and the concentration was confirmed as peroxide equivalents with a Palintest Photometer 5000 using the hydrogen peroxide HR test at 490 nm. The cultures were exposed to the sanitizer for 1, 2, 4, 8, and 16 min following procedures adopted from Cruz and Fletcher (2012). One milliliter of each culture was centrifuged (Eppendorf, 5424R) at 15,700 RCF for 5 min. The supernatant was removed, and cells washed with sterile water and re-suspended in 1 mL sterile water. Cell suspensions (50 μl) were dispensed into a 96-well plate and 50 μl of the sanitizer was added into each well. Plates were then left at room temperature for the designated contact time. To terminate the sanitizer reaction, 150 μl of neutralizer solution containing 5% egg yolk emulsion (Difco), 1% sodium thiosulfate (AnalaR, BDH, Chemicals Ltd., Poole, United Kingdom), and 0.5% Tween^®^ 80 (Spectrum, Gardena, CA, United States) in TSBYE was added to each well containing the bacterial culture and sanitizer. Neutralized suspensions were then serially diluted in BLEB up to 10^–15^, and survivors quantified using the MPN method.

### Heat Treatment

All *L. innocua* strains and the *LM* cocktail were exposed to 55°C for 15, 30, 60, and 120 min to investigate the inactivation of the isolates. These times were necessarily longer than usually used for thermal inactivation due to the lower than normal temperature being applied. An aluminum heating block (model D1105, Labnet Intl. Inc., Edison, NJ, United States) was placed in a water bath [type TC120, Grant Instrument (Cambridge Ltd.), Shepreth, United Kingdom]. Capped micro-tubes (0.6 mL thin-walled Axygen^TM^ MaxyClear Snaplock Microtubes, type MCT060R, Axygen Scientific Inc., NY, United States) containing 490 μl of BLEB were placed in the heating block which was then heated up to 55°C and left for 30 min to equilibrate to the test temperature before the cultures were added. Capped micro-tubes fitted with calibrated type-T thermocouples were used to measure the temperature and the temperature was measured at 1-s interval using a 1000 series Grant Squirrel meter/logger (Grant Instruments Ltd., Cambridge, United Kingdom). When the media had equilibrated at the desired test temperature, 10 μl of 48-h 30°C cultures was added to each micro-tube and mixed three times by aspiration using an automated pipette. There was a temperature drop (average = 2.64°C) while adding the cultures to the heated media and on average it took 2.23 min to return to the test temperature.

Bacterial counts were taken at T0 – untreated control and at Ts – specified time intervals at the test temperature. At each sampling interval, microtubes were placed on ice and were then serially diluted in BLEB in 96-well plates. Surviving cells were enumerated by the MPN method.

### Hurdle Treatments

All *L. innocua* strains used in the present study were tested individually in the hurdle treatments. Two hurdle treatments were conducted for the *L. innocua* individual strains and cocktails that were compared with the LM cocktails. These two hurdle treatments are referred to as “hurdle individual treatment” and “hurdle cocktail treatment.” In both hurdle treatments, hurdle 1 consisted of a combination of milder treatments, sequentially heat (55°C for 7.5 min) followed by UV-C (328 mJ/cm^2^) and sanitizer (10 ppm of Tsunami^TM^ 100 for 2 min). Hurdle 2 treatment (harsh treatment combinations) consisted of a combination of heat (55°C for 15 min) followed by UV-C (672 mJ/cm^2^) and sanitizer (10 ppm of Tsunami^TM^ 100 for 4 min). These hurdle combinations were selections as combinations that would likely inactivate many but not all of the target organisms, allow the reductions achieved for the *LM* cocktail, and the potential surrogates to be compared and contrasted. The procedures for each hurdle treatment were identical to the independent treatment procedures. The samples were heat-treated in capped micro-tubes, transferred into 6-well tissue culture plates for UV-C exposure, then transferred to a 96-well plate for sanitizer treatment, and finally neutralized. The samples were then serially diluted and the survivors were enumerated using the MPN method.

For the hurdle cocktail treatment, cocktails of *L. innocua* and LM cocktail were compared. The *L. innocua* strains used in the cocktails were selected based on their individual responses in comparison to the *LM* cocktail in the individual treatments conducted previously. The cocktails selected were (1) PFR 05A07 and 05A11; (2) PFR 05A11 and 16D08; (3) PFR 05A07 and 16D08; and (4) PFR 05A07, 05A11, and 16D08. These *L. innocua* cocktails were subjected to the hurdle 1 and 2 treatments as described above.

### Statistical Analysis

Each independent experiment was carried out twice while the hurdle combination experiments were carried out three times, each using each individual strain of *L. innocua* and the *LM* cocktail. The calculated MPNs were log-transformed, log_10_ reductions were calculated and the variance was stabilized. The standard errors of the mean were calculated and one-way analysis of variance (ANOVA) (Genstat Version 17, 2016) where the *p*-value of <0.5 was considered significant, and a *post hoc* Fisher’s least significant differences (LSDs, *p* < 0.05) were performed. These parameters were used to compare individual *L. innocua* strains, the *LM* cocktail, and *L. innocua* cocktails, and were presented graphically as LSD bars.

## Results

### Genotyping and Selection of *Listeria* Strains

The *Listeria* strains were genotyped by PFGE to select the candidate to be used in the inactivation treatments using *Asc*I and *Apa*I enzymes. Figures 1A,B represent the PFGE patterns of the *L. monocytogenes* and *L. innocua*. Nine genetically dissimilar *L. innocua* and 11 *L. monocytogenes* strains (combined in a cocktail, referred to as *LM* cocktail for convenience throughout the manuscript) were compared by treating them with UV-C, sanitizer, and heat, and a combination of these three. Figure 1A shows the dendrogram of the 10 *L. innocua* strains selected (based on at least 35% genetic dissimilarity) from 19 isolates, while Figure 1B shows the dendrogram of the *L. monocytogenes* strains selected for the cocktail.

### UV-C Treatment

**FIGURE 2:**
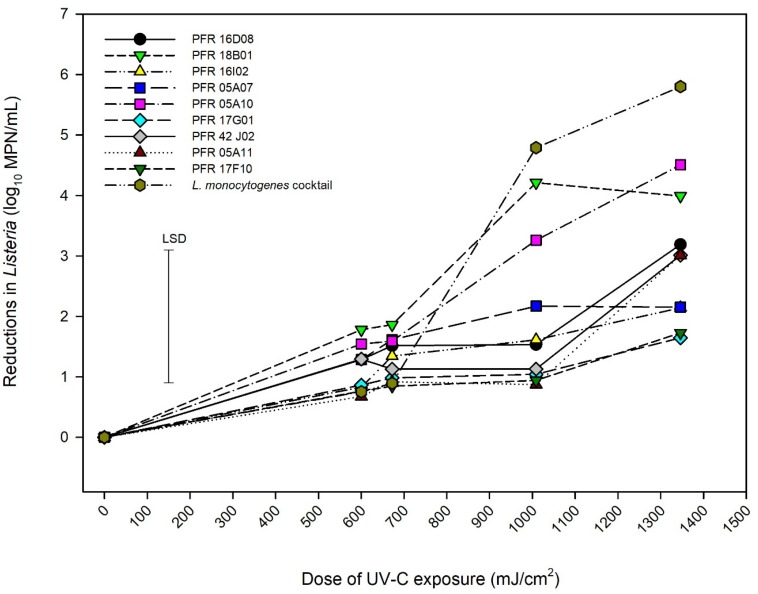
Reductions in numbers of *Listeria innocua* and of organisms in a *Listeria monocytogenes* cocktail when exposed to UV-C radiation. Error bar = least significant difference (*p* = 0.05, *n* = 2).

A non-thermal inactivation procedure, UV-C (245 nm) was used to investigate the effect of UV-C exposure on the survivability of various strains of *L. innocua* and the *LM* cocktail. Figure 2 represents the behavior of different strains of *L. innocua* and the LM cocktail. ANOVA showed that 5% of the variance was due to the irradiance while 20% was due to the strain (Height × strain) while the 25% residual variance would be accounted for by the differences in replicate experiments. Increasing the dose of UV-C exposure resulted in an increase in killing effect although reductions in counts were minor for some strains (e.g., PFR 17G901 and PFR 17F10). At exposure doses of 600 and 672 mJ/cm^2^, all strains responded in a similar manner with log_10_ reductions ranging from 0.5 to 1.75. As the dose was increased to >1000 mJ/cm^2^, different responses were observed for different strains. The *LM* cocktail had the greatest reductions in counts and only strains PFR 05A10 and PFR 18B01 had similar responses to that of the *LM* cocktail while the other strains clustered and were more resistant.

### Sanitizer Treatment

**FIGURE 3:**
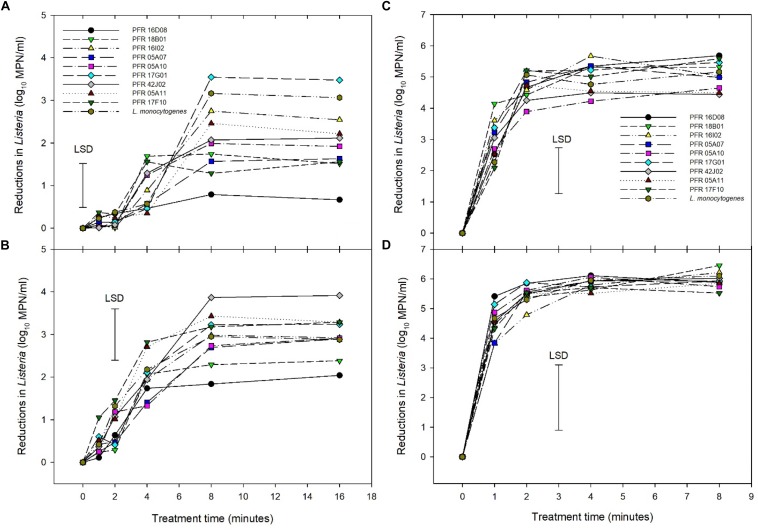
Reductions in numbers of *Listeria innocua* and of organisms in a *Listeria monocytogenes* cocktail during exposure to Tsunami^TM^ 100 at 10 ppm **(A)**, 20 ppm **(B)**, 40 ppm **(C)**, and 80 ppm **(D)**. Error bars = least significant differences (*p* = 0.05, *n* = 2).

Another non-thermal inactivation treatment employed was sanitizer (Tsunami^TM^ 100). Figures 3A–D show the pattern of reductions in *Listeria* count when treated with Tsunami^TM^ 100 at different peroxide equivalent concentrations of 10, 20, 40, and 80 ppm, respectively. ANOVA showed that sanitizer concentration and exposure time accounted for 88% of the variance compared to just 4% for the strains while the variation between replicate experiments would be responsible for the 8% residual variance. As expected, increasing contact time between the bacteria and Tsunami^TM^ 100 led to increased kill. No reductions in counts were observed during the first 2 min of exposure to 10 ppm Tsunami^TM^ 100 whereas reductions started to be observed during the first minute at the higher concentrations. Maximum inactivation was achieved by 8 min at concentrations of 10 and 20 ppm while this was mostly achieved within 2 min at 40 and 80 ppm. Increasing concentrations gave increasing maximum reductions in *Listeria* numbers. All strains behaved similarly to each other on exposure to 40 and 80 ppm Tsunami^TM^ 100 while significant differences were observed between strains at 10 and 20 ppm. At 10 ppm the *LM* cocktail was more sensitive to Tsunami^TM^ 100 than many of the *L. innocua* strains with PFR 16D08, PFR 17F10, PFR 05A07, and PFR 18B01 being significantly more resistant than *L. monocytogenes* after 8- and 16-min exposure. At 20 ppm, just 05A12 showed significantly more resistance after 4 min exposure but not at other time periods. *L. innocua strains* PFR 16I02 and PFR 17G01 always responded in a similar manner to the *LM* cocktail.

### Heat Treatment

**FIGURE 4:**
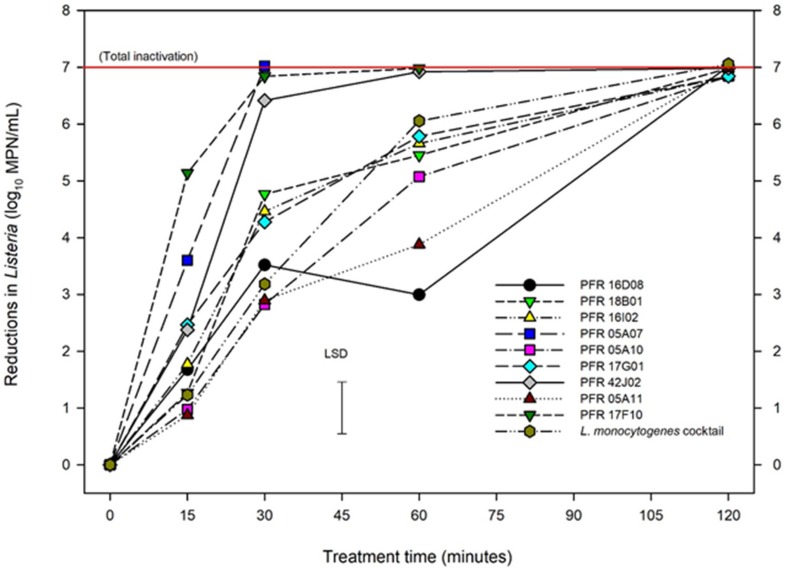
Reductions in numbers of *Listeria innocua* and of organisms in a *Listeria monocytogenes* cocktail at 55°C. Error bar = least significant difference (*p* = 0.05, *n* = 2).

Mild heat was employed as thermal inactivation procedure to assess the *Listeria* strains. Figure 4 represents the responses of different strains of *Listeria* to heat treatment which shows log_10_ reductions of *Listeria* during heat treatment at 55°C, over time up to 120 min. ANOVA showed less variation between repeat experiments for the heat treatments with temperature and time accounting for 76% of the variance, strain for 16%, and experiment to experiment variation just 3%. The *LM* cocktail showed log_10_-linear reductions up to 6 log_10_ MPN/mL at 60 min and then was totally inactivated by 120 min, as were all *L. innocua* strains. Different *L. innocua* strains had significantly different responses to heat treatment. Strains PFR 17F10, PFR 05A07, and PFR 42J02 were all substantially more heat sensitive than the *LM* cocktail with total inactivation occurring within 30 min. PFR 17G01 also showed significantly more heat sensitivity than the *LM* cocktail after 15 and 30 min of heat treatment but by 60 min its inactivation rate was reduced, resulting in a total inactivation very similar to the *LM* cocktail. Similarly, none of the other *L. innocua* strains consistently matched the inactivation rates of the *LM* cocktail. While all showed similar reductions at 15 min, two strains (PFR 18B01 and PFR 16I02) showed significantly more reduction at 30 min while the other three (PFR 05A10, PFR 05A11, and PFR 16D08) showed significantly less reduction at 60 min. The most similar was PFR 05A10 which had similar sensitivity to heat after 15 and 30 min and was only slightly more resistant than the *LM* cocktail after 60 min with a log_10_ reduction of 5.07 compared with a 6.06 log_10_ MPN/mL for the *LM* cocktail (LSD = 0.914 log_10_ MPN/mL).

### Hurdle Treatments

**FIGURE 5:**
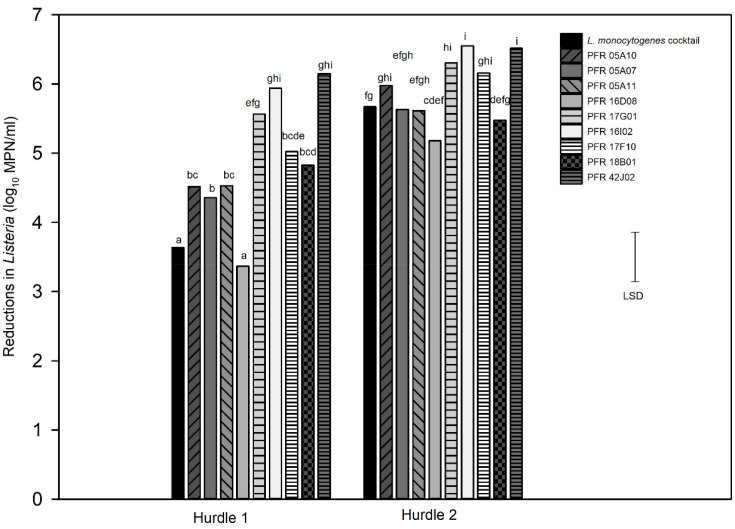
Hurdle individual treatments: reductions in numbers in individual *Listeria innocua strains* and a *Listeria monocytogenes* cocktail in response to hurdle treatments: heat (55°C) for 7.5 min, UV-C at 328 mJ/cm2, Tsunami^TM^ 100 at 10 ppm for 2 min (Hurdle 1) and heat (55°C) for 15 min, UV-C at 672 mJ/cm2, Tsunami^TM^ 100 at 10 ppm for 4 min (Hurdle 2). Error bar = least significant difference (*p* = 0.05, *n* = 3). The letters above the bars indicate the similarity and/or difference in reductions between the strains for example: “a” and “a” are similar to each other and “bc” and “bc” are similar to each other or do not show significant difference in reduction between those respective strains the bars stand for.

**FIGURE 6:**
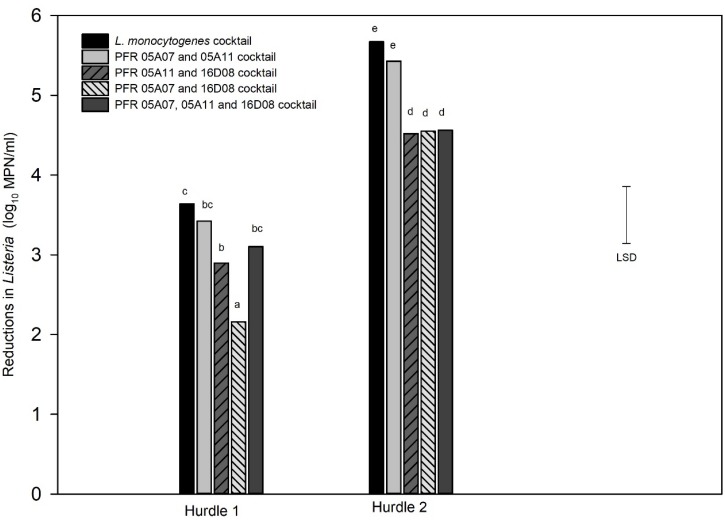
Hurdle cocktail treatments: Log_10_ MPN reductions in cell numbers of *Listeria innocua* cocktails and *L. monocytogenes* cocktail in response to hurdle treatments: heat (55°C) for 7.5 min, UV-C at 328 mJ/cm2, Tsunami^TM^ 100 at 10 ppm 2 min (Hurdle 1) and heat (55°C) for 15 min, UV-C at 672 mJ/cm2, Tsunami^TM^ 100 at 10 ppm 4 min (Hurdle 2). Error bar = least significant difference (*p* = 0.05, *n* = 3). The letters above the bars indicate the similarity and/or difference in reductions between the strains for example: “bc” and “bc” are similar to each other or do not show significant difference in reduction between each other and “d”s indicate similar reductions between those respective cocktails.

For the combination treatment (hurdle), two hurdle treatments were conducted for *L. innocua:* one for individual strains (hurdle individual treatment) and the other for *L. innocua* cocktails (hurdle cocktail treatment) that were compared with the LM cocktails. Figure 5 shows the log_10_ reductions for the nine individual *L. innocua* strains and the *LM* cocktail tested against the hurdle treatments. Figure 6 shows the log_10_ reductions for different combinations of *L. innocua* cocktails compared to that of the *LM* cocktail in the hurdle treatments.

When tested individually, all but one strain (PFR 16D08) gave significantly higher log_10_ reductions in hurdle 1. In hurdle 2, PFR 05A07, 05A11, and 18B01 responded very similarly to the *LM* cocktail but only PFR 17G01, 16I02, 17F10, and 41J02 were significantly different, all being more sensitive to the hurdle treatments with higher reductions. Only PFR 16D08 did not differ significantly to the *LM* cocktail in its response to both hurdle combinations (Figure 5) but this strain was also substantially more resistant to all the treatments when applied individually (Figures 2–4).

Cocktail combinations of three *L. innocua* strains (PFR 16D08, 05A07, and 05A11) that responded similarly to the *LM* cocktail were also subjected to the hurdle treatments (Figure 6). In both hurdle treatments, the *L. innocua* cocktail containing PFR 05A07 and 05A11 was not significantly different from that of the *LM* cocktail. There was also no significant difference in the response of the cocktail of the three strains (PFR 05A07, 05A11, and 16D08) to hurdle 1 but in all other instances, the *L. innocua* cocktail was significantly more resistant to the hurdle treatments than the *LM* cocktail. *L. innocua* strains with similar responses to the *LM* cocktail are summarized in Table 1.

**TABLE 1:** Summary of the *Listeria innocua* strains that responded similarly to the *L. monocytogenes* cocktail under treatment conditions that differentiated the inactivation responses of *L. innocua* strains from each other.

**Treatment**	**Treatment conditions**	***Listeria innocua* strains showing similar responses (*p* < 0.05) to those of the *LM* cocktail**
UV-C	1346 and 1009 mJ/cm^2^	PFR 05A10 and PFR 18B01
Tsunami^TM^ 100 (10 ppm)	4 min	All except PFR 18B01
Tsunami^TM^ 100 (10 ppm)	8 and 16 min	PFR 17G01, PFR 16I02, PFR 05A11, PFR 42J02, and PFR 05A10
Tsunami^TM^ 100 (40 ppm)	2 min	All except PFR 17F10
Heat (55°C)	15 min	All except PFR 17F10 and 05A07
Heat (55°C)	30 min	PFR 05A11, PFR 05A10, and PFR 16D08
Heat (55°C)	60 min	PFR 17G01, PFR 16I02, and PFR 18B01
Hurdle 1	55°C (7.5 min), 10 ppm Tsunami^TM^ 100 (2 min), and UV-C (328 mJ/cm^2^)	PFR 16D08, cocktail of PFR 05A07 and PFR 05A11, cocktail of PFR 16D08, PFR 05A07, and PFR 05A11
Hurdle 2	55°C (15 min), 10 ppm Tsunami^TM^ 100 (4 min), UV-C (328 mJ/cm^2^)	PFR 05A07, PFR 05A11, PFR 05A10, PFR 18B01, PFR 16D08, PFR 17F10, cocktail of PFR 05A07, and PFR 05A11

*The strains were compared based on the least significant differences (*p* = 0.05) for the individual or hurdle combination treatments.*

## Discussion

The aim of the present study was to select suitable strain(s) of *L. innocua* to be used as non-pathogenic surrogates for *L. monocytogenes* in the laboratory and pilot-scale process studies. This meant identifying a surrogate whose responses matched those of an *LM* cocktail as closely as possible. Some of the criteria that were considered in selecting a suitable surrogate were: a surrogate being more sensitive than LM could result in mild treatments being promoted which would not assure food safety, while being more resistant could result in selecting a harsh treatment that may have higher costs and could cause more damage to the product than necessary and, if no surrogate was found to totally match the pathogen then the aim was to choose one that was slightly more resistant so that food safety would be assured. Independent experiments were carried out with 10 *L. innocua* strains and a cocktail of 11 *L. monocytogenes* strains isolated from New Zealand fresh produce using treatments suitable for fruit or vegetables that were to be eaten raw: UV-C, sanitizer, and heat (Figures 2–4).

The germicidal effect of UV-C irradiation has long been known and has been employed widely to inactivate indicator organisms and pathogens in fresh produce, environmental biofilms, food products, and RTE food products (Chang et al., 1985; Bialka and Demirci, 2008; Gailunas et al., 2008; Keklik et al., 2008; Sommers et al., 2009; Rajkovic et al., 2010; Bernbom et al., 2011; Schenk et al., 2011; Liao et al., 2017). The UV-C spectrum range of 250–270 nm is strongly absorbed by microorganisms and is considered to be the most lethal range of wavelengths, with 262 nm being the peak germicidal wavelength (Gurzadyan et al., 1995). Oteiza et al. (2005) found that UV exposure was an efficient method to treat fruit juice contaminated with *Escherichia coli* O157: H7 investigated *L. monocytogenes* and *L. innocua* using UV-C at 254 nm and found that a dose of 4 J/cm2 achieved a 0.37 log_10_ reduction in *L. innocua* and a 1.93 log_10_ reduction in *L. monocytogenes* on frankfurters. They attributed the difference to the surface topography of the meats and the ingredients of the frankfurters. Similarly, sanitizers have been employed as a common method for disinfection, particularly of processing environments, equipment, and wash waters. One such post-harvest sanitizer is Tsunami^TM^ 100 (Ecolab Inc., MN, United States), a mixture of acetic acid, peroxyacetic acid, and hydrogen peroxide (Banach et al., 2015). It has been shown to be an effective treatment to inactivate spoilage and pathogenic bacteria including *E. coli*, *Listeria*, and *Salmonella* with its efficacy depending on the contact time, temperature, and concentration (Lin et al., 2002; Alvaro et al., 2009). Yet another emerging inactivation technology for improving the quality of food and its shelf life is hurdle technology. This concept of applying a series of hurdles in the post-harvest period was developed to address consumer demands for fresh and safe produce. It is an intelligent combination of more than one post-harvest antimicrobial treatment to secure microbial safety and stability, retaining the organoleptic and nutritional quality of food products and these strategies have been used for meat, fish, milk, and vegetables for years (Leistner, 2000). Some of the more recent hurdle technologies include nano-thermosonication, ultrahigh pressure, photodynamic inactivation, modified atmosphere packaging of both non-respiring and respiring products, edible coatings, ethanol, and products to control Maillard reactions. These have been gaining popularity in recent years [reviewed by Gayan et al. (2012) and Aditya and Nida (2015)].

Montgomery and Banerjee used pulsed UV light (PUV) for 10 and 20 s to treat *L. monocytogenes* and *E. coli* biofilms on the surfaces of lettuces. It was observed that longer PUV exposure time and shorter light source distance to the sample (20 s, 4.5 cm) resulted in a significant viable cell reduction of both pathogens compared to shorter exposure time and longer light source distance (10 s, 8.8 cm). Similarly, in the present study, increase in reduction rates was observed with increased exposure time and shorter light source distance. On average all the *L. innocua* strains were more resistant to UV-C than the cocktail of 11 diverse strains of *L. monocytogenes* with all strains except PFR 18B01 and PFR 05A10 being significantly more resistant at doses of over 1000 mJ/cm^2^. This suggests that in general *L. innocua* is more resistant to UV-C than *L. monocytogenes*. Previous studies have used UV-C to inactivate *L. monocytogenes* from meat, processed food products, and fresh produce (Gailunas et al., 2008; Keklik et al., 2009; Sommers et al., 2009; Rajkovic et al., 2010; Bernbom et al., 2011; Liao et al., 2017; Mikš-Krajnik et al., 2017). However, these studies cannot be compared directly to the current study due to the differences in matrices, the dose of UV-C, and experimental procedures. Furthermore, it is acknowledged that the bacterial culture medium is quite different from the real products including processed and raw food products in respect to their nature and physicochemical properties, where these procedures may not be directly applicable. However, in saying that, horticultural produce such as fruits and vegetables, their surfaces are harbored by microbes and their surfaces or skin surfaces are washed with sanitized water or UV treated to inactivate the microbial load on their surfaces. This research is based on a number of unpublished data on fresh produce preservation techniques that have used heat, UV, sanitizers, and hurdles to reduce microbial load on the surfaces of fruits in New Zealand. A patent requisition had been submitted in 2001 (Stanley, 2001).

Similarly, in the food industry, sanitizers and cleaning agents have been used in cleaning regimes for decades to reduce microbial contamination, thereby improving product shelf life and food safety. Previous studies have investigated the inactivation efficiency of Tsunami^TM^ 100 (acetic acid, peroxyacetic acid, and hydrogen peroxide) in different fresh produce (Larry et al., 2004; Cruz and Fletcher, 2012; Karl et al., 2014; Banach et al., 2015; Eva et al., 2015). These studies indicated that peroxyacetic acid-based sanitizers are effective at lower concentrations compared to other sanitizers and were effective at killing *Listeria* spp. and their biofilms. In this study, as expected, the inactivation efficiency of Tsunami^TM^ 100 increased with increased concentration and time of exposure. The United States Environmental Protection Agency (US EPA) has recommended 30–80 ppm for 45 s to yield an effective inactivation of non-pathogenic spoilage organisms on fresh produce and 30 min for soil pathogens (Ecolab, 2013). When applying Tsunami^TM^ 100 to *L. innocua* cultures suspended in clean water, rapid reductions in numbers at 40 and 80 ppm were observed. Maximum inactivation (4 and 5 log_10_ reductions at 40 and 80 ppm, respectively) was not observed until exposure times exceeded 2 and 1 min, respectively (Figures 3C,D). The lower concentrations of 10 and 20 ppm also gave increasing inactivation of most *L. innocua* strains with increasing contact time up to 8 min (maximum reductions all below 4 log_10_) after which further extension of contact time did not show any additional benefit (Figures 3A,B). In some of the more resistant strains, inactivation stopped after just 4 min (PFR 16D08 and PFR 18B01 at both 10 and 20 ppm and PFR 17F10 just at 10 ppm). At the lowest tested concentration of 10 ppm, just two *L. innocua* strains (PFR 16I02 and PFR 17G01) had very similar log_10_ reductions in numbers to that of the *LM* cocktail while the other strains were more resistant to sanitizers than the *LM* cocktail.

In the heat treatment, the survival rate decreased with extended heating time, which was expected and has been observed by other researchers (Murphy et al., 2004). All strains had been totally inactivated after 120 min at the relatively mild temperature of 55°C. Such an exposure time is unlikely to be practical as a post-harvest treatment and different strains responded differently to heat treatment after shorter exposure times.

In another thermal inactivation study evaluating *L. innocua* strains as surrogates, Friedly et al. (2008) used five different *L. innocua* strains M1, 5639, 5640, and 2745 (from Special *Listeria* Culture Collection, University of Wurzburg, Germany) and tested them against the higher temperatures (62.5–70°C) suitable for hamburger patties. They recommended using M1 as a surrogate as it had the “greatest” margin of safety. M1 is well characterized and has been used by other researchers for *Listeria* surrogate work around the world (Murphy et al., 2003; Friedly et al., 2008) and this strain has been used in thermal inactivation studies of various products in the laboratory environment Friedly et al., 2008. However, the temperatures used by Friedly et al. (2008) are too high for fresh produce due to its sensitivity to heat. The approach of using the organism with the greatest margin of safety (i.e., the most resistant) as a surrogate would be likely to lead to using processes that give unacceptable losses in quality much milder than those applied to milk and meat. In this lab-based study all *L. innocua* strains had significantly different (*p* < 0.05) responses to the *LM* cocktail in one or more of these individual treatments. Across the three treatments, PFR 05A10 was the most similar in its responses compared to the *LM* cocktail, only being significantly different at 55°C for 60 min (Table 1). This strain invariably gave responses that showed it to be either very similar or slightly more resistant to the individual treatments than the *LM* cocktail (lines below that of the cocktail in Figures 2–4). If a single strain were to be used to evaluate all the single treatments, this strain would be the best surrogate of those tested. However, for the sanitizer, although PFR 05A10 was not significantly different to the *LM* Cocktail, PFR 16I10 was consistently more similar to the *LM* cocktail than PFR 05A10 so this strain might be a better surrogate for studies on peroxyacetic acid-based sanitizer like Tsunami^TM^ 100. Like PFR 05A10, it was also slightly more resistant than the *LM* cocktail.

Given that *L. monocytogenes* can survive under harsh conditions and only mild treatments can be applied to fresh produce, inactivation of *L. monocytogenes* using a single treatment is unlikely to provide a post-harvest regime sufficient to achieve desired reductions in bacterial numbers. The concept of combined application of pathogen inactivation procedures has been accepted and widely used for the past decade. In the present study, it was found that each individual treatment (UV-C, Tsunami^TM^ 100 and heat) gave a different profile of *L. innocua* strains that responded most closely to the *LM* cocktail. This suggests that in a real-world scenario, each *L. innocua* strain will respond to UV-C, sanitizer, and heat differently and a cocktail of surrogates would be appropriate for use in inactivation studies. For example, strains PFR 18B01 and PFR 05A10 responded most similarly to UV-C exposure, as did the *LM* cocktail, whereas PFR 16I02 and PFR 17G01 responded most similarly to the sanitizer treatment at the lower concentration of 10 ppm and the strain PFR 05A10 was most similar in the heat treatment.

When the three treatments were combined into a series of hurdles, dramatically greater reductions in numbers were achieved for the individual strains (Figure 5) compared to when the single treatments were applied (Figures 2–4). In the milder hurdle combination (Hurdle 1) that was applied at about half the strength as in Hurdle 2, all strains suffered more than 3 log_10_ reductions with some reaching 6 log_10_ reductions. The UV-C, sanitizer, and heat treatments used in the Hurdles did not achieve significant reductions for any of the strains when applied as individual treatments but when combined as a hurdle combination, all strains suffered at least 5 log_10_ reductions in numbers. This demonstrated that these three treatments work synergistically. In general, the synergistic hurdle effect of the three combined treatments was mirrored in both the individual strains and the cocktails. Although hurdle technology has been applied to different fresh-cut-produce and food products (Gayan et al., 2012; Kumar et al., 2015; Singh et al., 2015), the present laboratory-based hurdle study cannot be compared with these studies as they involved different treatments as combinations including spray washing, essential oils, high-pressure processing, sonication, and other as suited for different food products.

Several strains including PFR 16D08 responded to Hurdle 2 in a similar manner to the *LM* cocktail but only PFR 16D08 was as resistant to the mild Hurdle 1 treatment as the *LM* cocktail. PFR 05A10 that responded similarly to the *LM* cocktail when the treatments were applied individually was significantly more sensitive when they were applied as a combination in Hurdle 1. If such a strain were to be used as a surrogate, it could result in researchers underestimating the effect of a treatment against *L. monocytogenes* and potentially leading to the sale of the unsafe product. While PFR 16D08 on its own could be selected as a surrogate for this combination of treatments, when the treatments were applied individually, PFR 16D08 was usually much more resistant than the *LM* cocktail. This was of concern as, when applied for studies on actual produce, it might be found necessary to apply some of the hurdles at even lower intensities that were applied in Hurdle 1. For example, some produce might be damaged at 55°C and holding product in such a treatment for 7.5 min might be too long for high-throughput industries. If the intensities of one or more of the treatments had to be reduced the combination system might respond more similarly to the single treatment. In this case, if PFR 16D08 were being used, it is likely that more intense treatments would be adopted than necessary to achieve target reductions in *L. monocytogenes*, and there is a danger of recommending treatment regimes, that may not be suitable for sensitive or highly perishable fresh produce, causing unacceptable damage to the produce and costing the industry more. Higher sanitizer concentrations might also be recommended, leading to unnecessary chemical residues.

The responses of three cocktail combinations of *L. innocua* to the two hurdle treatments were also tested (Figure 6). As with the individual treatments, the cocktails that included PFR 16D08 were consistently more resistant to the hurdle treatments than the *LM* cocktail. However, the responses of the cocktail of PFR 05A07 and PFR 05A11 were not significantly different from those of the *LM* cocktail. When challenged with Hurdle 2 as individual strains, PFR 05A07 and PFR 05A11 responded almost exactly the same as the *LM* cocktail although they were more sensitive to the Hurdle 2 combination (Figure 5). Both were more resistant to UV than the *LM* cocktail (Figure 2) so have the same potential limitations as PFR 16D08 in this regard. They were more similar in their response to the sanitizer (Figure 3) than PFR 16D08 and, although PFR 05A10 was very sensitive to heat inactivation, this was balanced by PFR 05A11 which was more similar in its response than PFR 16D08, particularly after 60 min exposure. Overall, from the data presented in this paper, when investigating individual and hurdle combinations to inactivate *L. monocytogenes*, it is recommended that using not an individual as surrogate but the cocktail of PFR 05A08 and PFR 05A11 although others might prefer to use just PFR16D08.

To conclude, 9 *L. innocua* strains and 11 *L. monocytogenes* strains (combined in a cocktail) were compared by treating them with UV-C, sanitizer, and heat, and a combination of these three in an effort to select suitable surrogate candidate(s). The results indicated that each *L. innocua* strain responded differently. The hurdle treatment produced a synergistic inactivation that had a significant reduction in the survival rates for the individual species and the cocktails. This study indicated that a cocktail of PFR 5A08 and PFR 5A11 strains may serve as a good surrogate for fresh produce thermal, UV-C, sanitizer, and hurdle studies with a significant safety margin. Testing these strains in different food matrices and post-harvest hurdle treatment regimes will provide insights into recommended heating times for inactivating *Listeria* spp. and/or *L. monocytogenes* in food products and recommended dose–time combinations for inactivating *Listeria* in fresh produce. It is acknowledged that this study did not investigate the response of individual *L. monocytogenes* strains to different treatment regimes due to resources constraints which would have been very helpful to better understand the variabilities within *L. monocytogenes* strains. The study highlights that it is important to test more than one surrogate strain to obtain an effective inactivation regime in food products as different strains exhibit different responses to inactivation procedures. The current study was carried out in laboratory media, but future studies should test different food matrices for validating the *L. innocua* strains to be used as potential surrogates.

## Data Availability Statement

The raw data supporting the conclusions of this manuscript will be made available by the authors, without undue reservation, to any qualified researcher.

## Author Contributions

VM did the experiment and wrote the manuscript. RW participated in the experiment and contributed to the manuscript sections “Results” and “Materials and Methods.” LH did the initial experiment for the research work. GS contributed to the experiment and in the review of the manuscript. GF contributed to the study design and manuscript writing.

## Conflict of Interest

All authors were employed by company The New Zealand Institute for Plant & Food Research Limited.
