# Supplementary material for: Identifying Suitable Listeria innocua Strains as Surrogates for Listeria monocytogenes for Horticultural Products
Source: Front Microbiol. 2019 Oct 9;10:2281. doi: 10.3389/fmicb.2019.02281 (PMC6794387; doi:10.3389/fmicb.2019.02281)
Supplement: Supplementary file 2 [file Data_Sheet_1.DOCX]

# **Identifying suitable *Listeria innocua* strains as surrogates for *Listeria monocytogenes* for horticultural products**

Vathsala Mohan^1^, Reginald Wibisono^1^, Lana de Hoop^1†^, Graeme Summers^1^ and Graham C Fletcher^1*^

1: Food Safety and Preservation Team, The New Zealand Institute for Plant & Food Research Limited, Auckland, NZ.

†: Current address: MediaCom, London, UK.

*: Corresponding author: Email: [graham.fletcher@plantandfood.co.nz](mailto:graham.fletcher@plantandfood.co.nz)

Supplementary material

Figure 1A and 1B

Original gel images


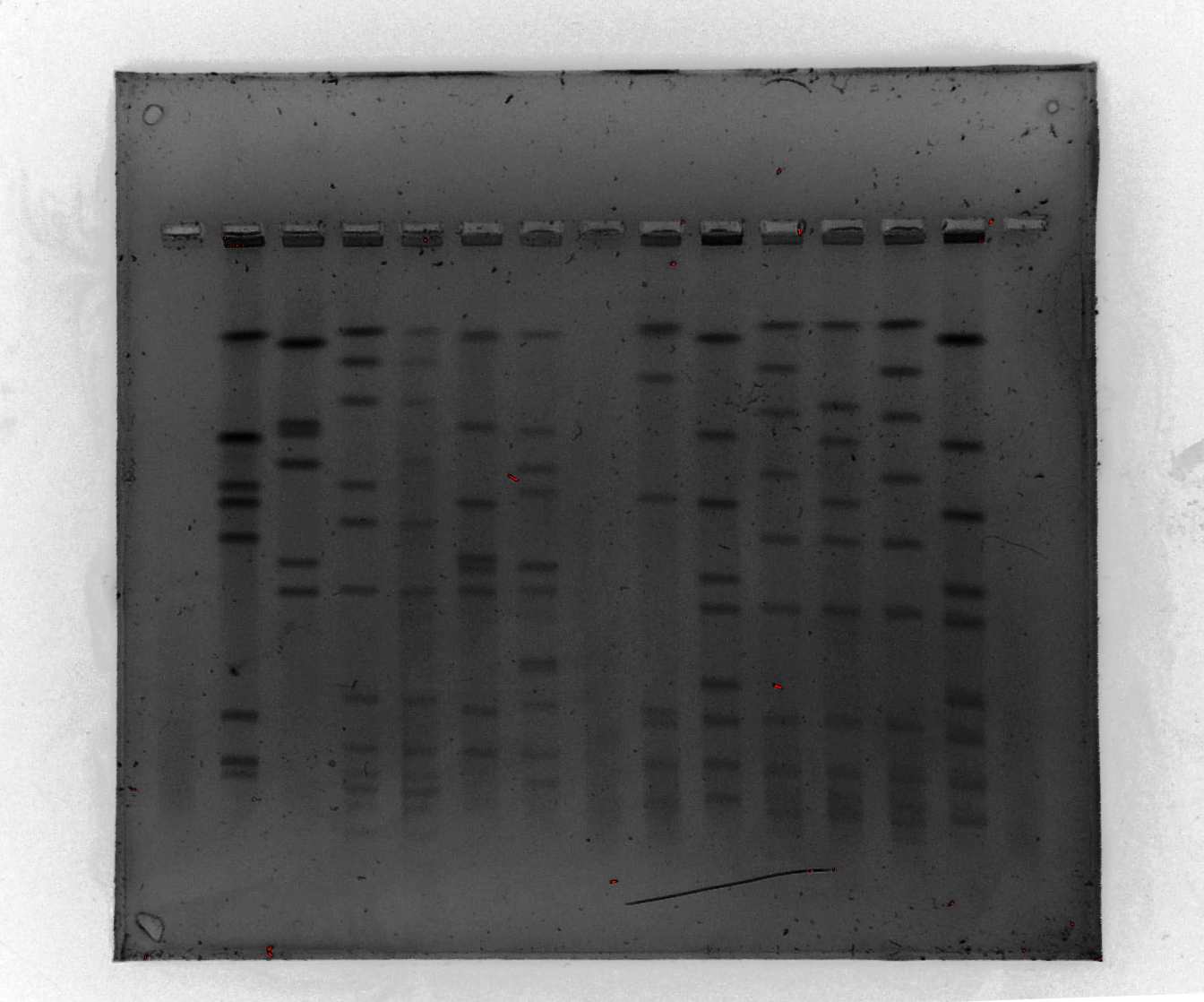


(A) Pulse Field Gel Electrophoresis of *Listeria innocua* isolates – Gel 1


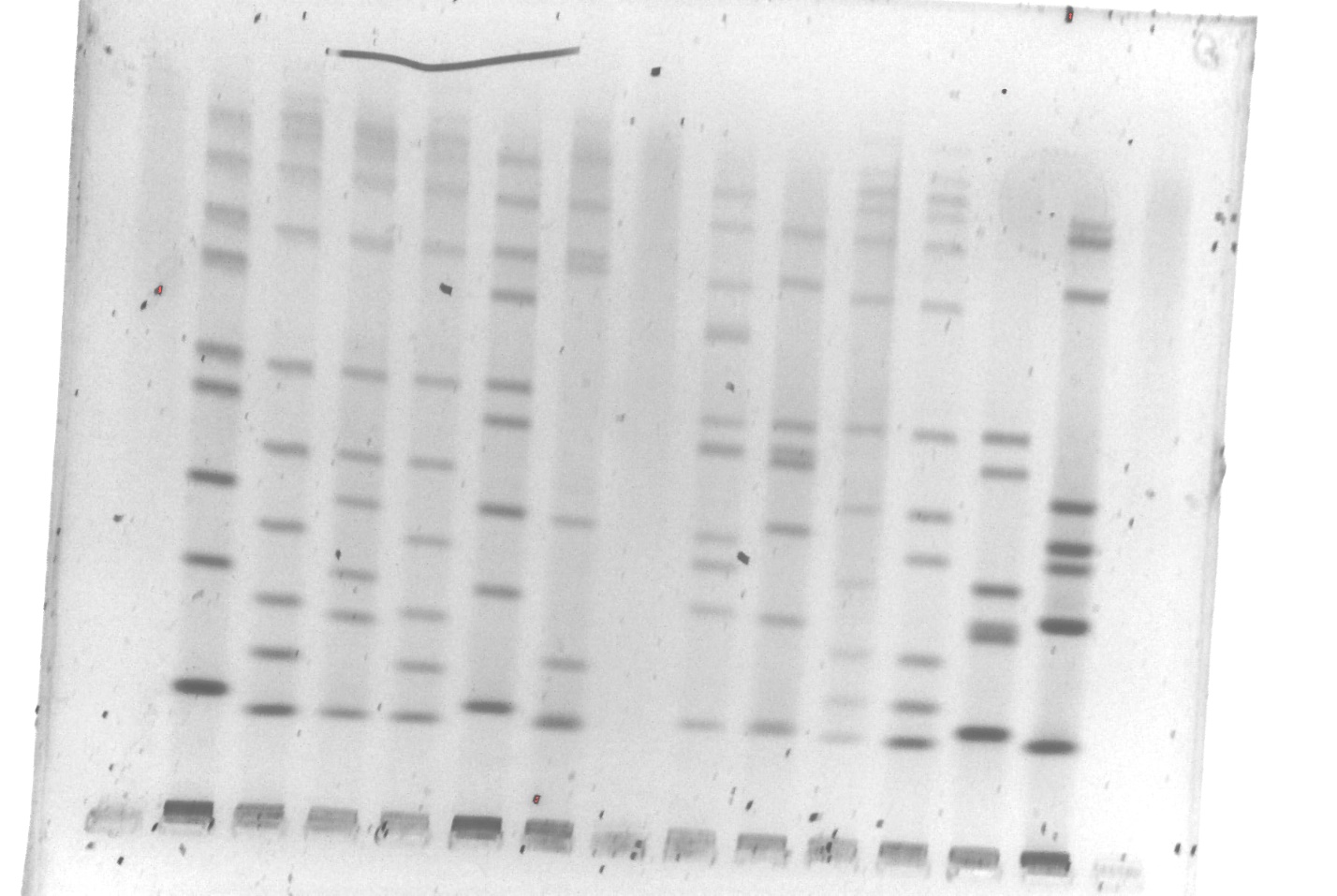


(A) Pulse Field Gel Electrophoresis of *Listeria innocua* isolates – Gel 2





(A) Pulse Field Gel Electrophoresis of *Listeria innocua* isolates -Gel 3


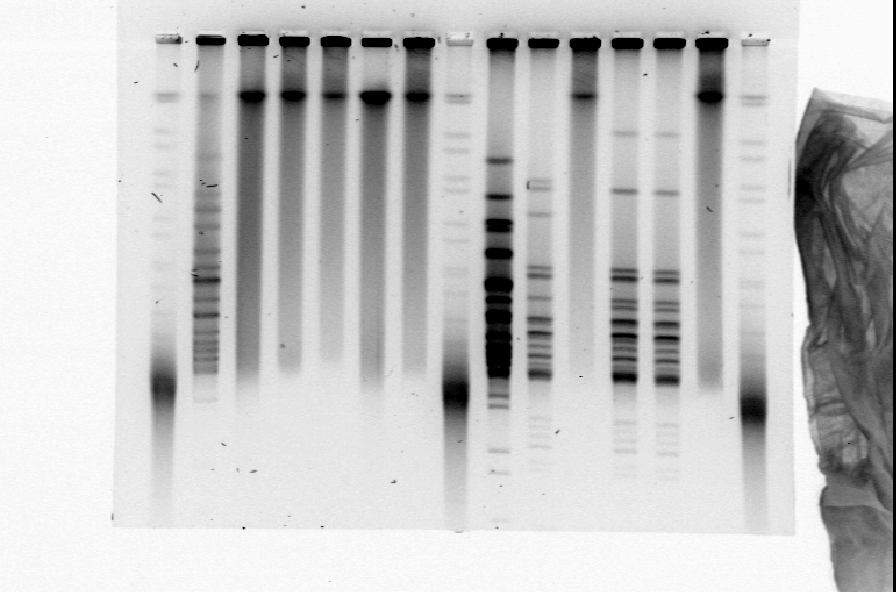


(B) Pulse Field Gel Electrophoresis of *Listeria monocytogenes* isolates -*Apa*I Gel 1


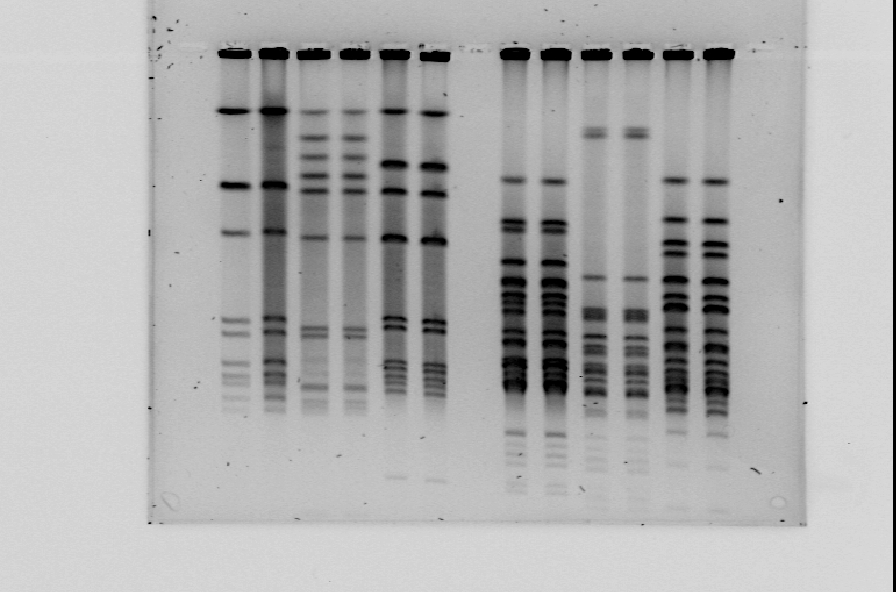


(B) Pulse Field Gel Electrophoresis of *Listeria monocytogenes* isolates -ApaI Gel 2


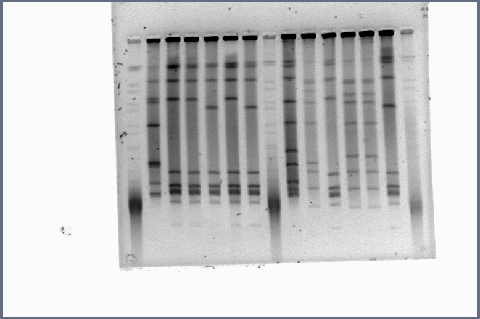


(B) Pulse Field Gel Electrophoresis of *Listeria monocytogenes* isolates -AscI Gel 3
